# Performance of urine DNA Neuritin 1 methylation test for urothelial carcinoma detection

**DOI:** 10.1186/s43556-025-00245-y

**Published:** 2025-02-08

**Authors:** Yucai Wu, Yanqing Gong, Liqun Zhou, Abai Xu, Xuesong Li

**Affiliations:** 1 Department of Urology, Peking University First Hospital, Institute of Urology, Peking University; National Urological Cancer Center; Urogenital Diseases (Male) Molecular Diagnosis and Treatment Center, Peking University, 8 Xishiku Street, Beijing, 100034 China; 2 Department of Urology, Zhujiang Hospital, Southern Medical University, Guangzhou, Guangdong China

**Dear Editor,**

Urothelial carcinoma (UC) includes urothelial carcinoma of the bladder (UCB) and upper tract urothelial carcinoma (UTUC), with UCB accounting for 90% of cases [1]. Among patients with high-grade (HG) non-muscle-invasive bladder cancer (NMIBC), 75% experience recurrence within 10 years, with 41% relapsing without disease progression and 33% progressing to muscle-invasive disease [2]. The prognosis for NMIBC that progresses to muscle-invasive disease is poor, highlighting the need for early and thorough investigation of this high-risk group. Currently, several screening methods are available, including urine cytology (25%−35% sensitivity) and UroVysion fluorescence in situ hybridization (FISH, 60%−80% sensitivity). Both methods have limitations in sensitivity and accuracy, especially for early-stage or low-grade (LG) tumors [3]. Cystoscopy and ureteroscopy with biopsy, considered the gold standards, are invasive procedures with low patient compliance. Recently, urine DNA methylation has emerged as a promising, non-invasive diagnostic tool. Specific methylation patterns in urinary cells have been identified, including those detected by the Bladder EpiCheck urine test, which shows 68.2% sensitivity and 88.0% specificity for monitoring recurrence in NMIBC patients [4]. While numerous studies have investigated urine DNA methylation for UC diagnosis, most focus on multiple targets or combine it with gene mutation detection. Single-target detection methods often demonstrate lower sensitivity, which poses challenges for clinical application.

Neuritin 1 (*NRN1*), an extracellular protein anchored to the cell membrane, was initially identified as a key factor in neuroprotection and regeneration [5]. Recent studies have shown that the methylation status of *NRN1* is associated with tumor progression, and that *NRN1* influences tumor growth through the Notch signaling pathway [5]. This study reports the development of a single-target *NRN1* methylation detection method and its clinical validation in a double-blind, multicenter, prospective study (Fig. 1a). The model development phase was initially conducted in a case–control cohort (*n* = 98). Subsequently, the clinical validation phase was carried out in two independent prospective clinical cohorts (*n*_1_ = 245, *n*_2_ = 110). Detailed information on participant recruitment and laboratory procedures is available in the Supplementary Methods (Supplementary File 1).

**Fig. 1:**
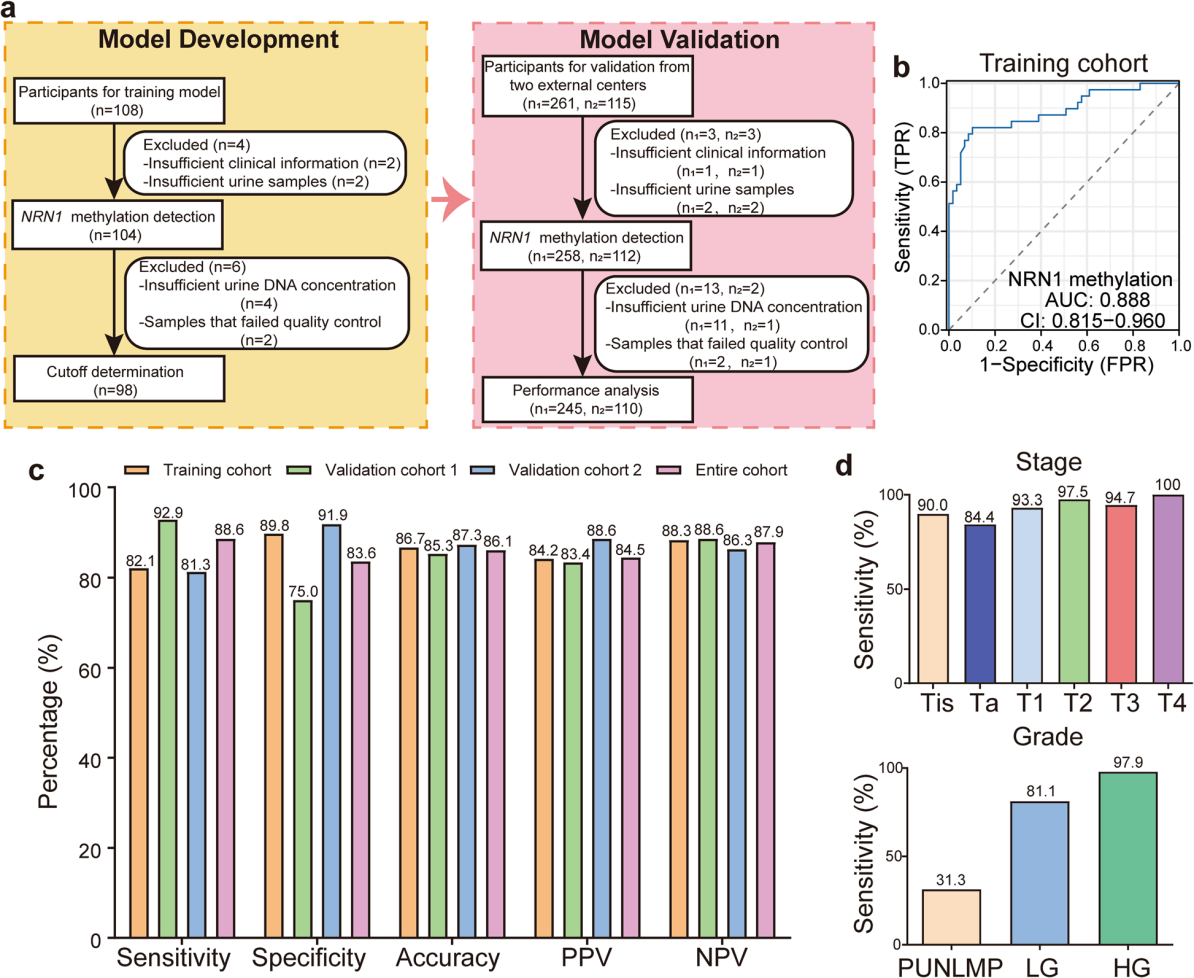
Development and validation of the *NRN1*^me^ test. **a** Workflow of the study design. **b** ROC curves and corresponding AUCs for *NRN1* methylation in diagnosing UC from controls samples were analyzed in the training cohort. **c** The sensitivity, specificity, accuracy, PPV, and NPV of the *NRN1*^me^ test were determined in the training, validation, and entire cohorts using the specified cutoff value. **d** The sensitivity of the *NRN1*^me^ test for UC, stratified by stage and grade. UC, urothelial carcinoma; ROC, receiver operating characteristic; AUC, area under the curve; PUNLMP, papillary urothelial neoplasm of low malignant potential; LG, low grade; HG, high grade; PPV, positive predictive value; NPV, negative predictive value

*NRN1* methylation (*NRN1*^me^) levels were significantly higher in the UC group compared to the control group, with this difference being more pronounced in tumors with higher pathological stages (T1-T4) (*p* < 0.0001, Fig. S1a). Additionally, *NRN1*^*me*^ levels were higher in HG UC compared to LG UC (*p* < 0.0001, Fig. S1a). Receiver operating characteristic (ROC) analysis demonstrated that *NRN1*^me^ could differentiate UC from all control groups in the training cohort, with an area under the ROC curve (AUC) of 0.888 (95% CI: 0.815–0.960, Fig. 1b). In the training cohort, a Delta Ct value of 10.18 yielded the highest Youden index. For clinical convenience, the cutoff value was set at 10. The *NRN1*^me^ test demonstrated a sensitivity of 82.1% for UC patients, with an overall specificity of 89.8% (Fig. 1c). In the clinical validation phase, participants from two additional centers were included, with validation cohort 1 consisting of 245 participants and validation cohort 2 consisting of 110 participants. The sensitivity of the *NRN1*^me^ test for detecting UC in validation cohort 1, cohort 2, and the overall cohort was 92.9%, 81.3%, and 88.6%, respectively; the specificity was 75.0%, 91.9%, and 83.6%, respectively (Fig. 1c). Additionally, the accuracy, positive predictive value (PPV), and negative predictive value (NPV) all exceeded 83% (Fig. 1c).

Notably, the *NRN1*^me^ test demonstrated excellent diagnostic performance in early-stage and LG UC. The detection rates increased with tumor stage and grade, showing sensitivities of 84.4%, 93.3%, 97.5%, 94.7%, and 100% for Ta, T1, T2, T3, and T4 tumors, respectively. The detection rate for carcinoma in situ was also 90% (Fig. 1d). Furthermore, the *NRN1*^me^ test exhibited sensitivities of 31.3%, 81.1%, and 97.9% for papillary urothelial neoplasm of low malignant potential (PUNLMP), LG, and HG tumors, respectively (Fig. 1d). For small tumors (≤ 1.5 cm), the sensitivity remained high at 86.8%, increasing with tumor size (Fig. S1b). Overall, the *NRN1*^me^ test proved to be accurate and robust across various clinical settings for UC patients, including those with early-stage, LG, or small tumors.

A comparative analysis of the *NRN1*^me^ test and common urinary tests, such as FISH and cytology, was conducted. In a subset of 70 patients who underwent both *NRN1*^me^ and FISH testing, the *NRN1*^me^ test demonstrated significantly higher sensitivity compared to FISH (92.5% vs. 81.1%) with comparable specificity (76.5% vs. 70.6%) (Fig. S1c). In patients with Ta tumors, the sensitivity of the *NRN1*^me^ test was three times higher than that of FISH (100% vs. 33.3%). Remarkably, the *NRN1*^me^ test showed a significant improvement in sensitivity for LG tumors compared to FISH (83.3% vs. 58.3%) (Fig. S1c). A total of 67 patients underwent both *NRN1*^me^ and cytology testing simultaneously. Although the specificity of the *NRN1*^me^ test was lower than that of cytology (70.6% vs. 100%), its sensitivity was twice as high (92.0% vs. 44.0%) (Fig. S1d). In Ta stage and LG tumors, the sensitivity of *NRN1*^me^ test is 1.3 times and 3 times higher than that of cytology, respectively (Fig. S1d). Indeed, this advantage was observed across subgroups of tumors with different stages and grades (Fig. S1d). Overall, compared to FISH and urine cytology, the *NRN1*^me^ test demonstrated significant advantages in sensitivity, particularly for early-stage and LG tumors.

Liquid biopsy is a method involving the sampling and analysis of body fluids, such as blood, primarily for tumor diagnosis or monitoring. In UC, liquid biopsies, including plasma circulating tumor DNA (ctDNA) and urinary tumor DNA (utDNA), have shown significant promise in diagnosis, prognosis, therapy response monitoring, and minimal residual disease detection. This study uses utDNA rather than ctDNA, which allows for more convenient and non-invasive sampling. Additionally, the methylation-specific PCR (MSP) detection method used in this study is technically straightforward, making it suitable for routine laboratory settings. Moreover, the single-gene *NRN1* methylation assay demonstrates good sensitivity, exhibiting higher detection sensitivity even for specimens with a low tumor burden. Compared to multi-gene panels or whole-genome sequencing, single-gene MSP detection is relatively cost-effective and significantly reduces the financial burden on patients. Lastly, the MSP detection process is relatively rapid, potentially shortening the diagnostic cycle and facilitating early clinical intervention. In contrast, methods such as multi-gene panels typically require more time for preparation and analysis.

In conclusion, we developed a urine assay utilizing the *NRN1*^me^ test for UC detection, validated in a multicenter cohort and demonstrating markedly higher sensitivity and specificity than urine cytology and FISH. Our assay used PCR-based techniques to identify *NRN1* methylation, offering a straightforward and cost-efficient approach. This novel test holds significant potential to transform UC diagnosis and management, enhancing patient outcomes while minimizing reliance on invasive procedures.

## Supplementary Information

Supplementary Material 1.

Supplementary Material 2.

## Data Availability

The datasets used and/or analyzed during the current study are available from the corresponding author upon reasonable request.

## References

[1] Miyazaki J, Nishiyama H. Epidemiology of urothelial carcinoma. International journal of urology : official journal of the Japanese Urological Association. 2017;24(10):730–4. 10.1111/iju.13376. 28543959 10.1111/iju.13376

[2] Chamie K, Litwin MS, Bassett JC, Daskivich TJ, Lai J, Hanley JM, et al. Recurrence of high-risk bladder cancer: a population-based analysis. Cancer. 2013;119(17):3219–27. 10.1002/cncr.28147. 23737352 10.1002/cncr.28147 PMC3773281

[3] Chen X, Zhang J, Ruan W, Huang M, Wang C, Wang H, et al. Urine DNA methylation assay enables early detection and recurrence monitoring for bladder cancer. J Clin Investig. 2020;130(12):6278–89. 10.1172/jci139597. 32817589 10.1172/JCI139597 PMC7685755

[4] Witjes JA, Morote J, Cornel EB, Gakis G, van Valenberg FJP, Lozano F, et al. Performance of the Bladder EpiCheck™ Methylation Test for Patients Under Surveillance for Non-muscle-invasive Bladder Cancer: Results of a Multicenter, Prospective. Blinded Clinical Trial Eur Urol Oncol. 2018;1(4):307–13. 10.1016/j.euo.2018.06.011. 31100252 10.1016/j.euo.2018.06.011

[5] Devitt L, Westphal D, Pieger K, Schneider N, Bosserhoff AK, Kuphal S. NRN1 interacts with Notch to increase oncogenic STAT3 signaling in melanoma. Cell Commun Signal. 2024;22(1):256. 10.1186/s12964-024-01632-8. 38705997 10.1186/s12964-024-01632-8 PMC11071257

