# Supplementary material for: Performance of urine DNA Neuritin 1 methylation test for urothelial carcinoma detection
Source: Mol Biomed. 2025 Feb 8;6:8. doi: 10.1186/s43556-025-00245-y (PMC11805734; doi:10.1186/s43556-025-00245-y)
Supplement: Supplementary file 1 — Supplementary Material 1. [file 43556_2025_245_MOESM1_ESM.docx]

**Performance of urine DNA Neuritin 1 methylation test for detecting urothelial carcinoma**

Yucai Wu, MD^1#^, Yanqing Gong, PhD^1#^, Liqun Zhou, MD^1*^, Abai Xu, MD^2**^, Xuesong Li, MD^1***^

1 Department of Urology, Peking University First Hospital; Institute of Urology, Peking University; National Urological Cancer Center; Urogenital Diseases (Male) Molecular Diagnosis and Treatment Center, Peking University, Beijing, China.

2 Department of Urology, Zhujiang Hospital, Southern Medical University, Guangzhou, Guangdong, China.

# Yucai Wu and Yanqing Gong contributed equally to this work.

Corresponding author: * Liqun Zhou, E-mail: zhoulqmail@sina.com

** Abai Xu, E-mail: lc96xab@163.com

*** Xuesong Li, E-mail: [pineneedle@sina.com](mailto:pineneedle@sina.com)

Department of Urology, Peking University First Hospital, 8 Xishiku Street, Xicheng, Beijing 100034, China (Xuesong Li, Tel. +010-83575101)

**Methods**

**Patients and samples**

This study was approved by the ethics committees of participating centers prior to its commencement. All participants were recruited and provided written informed consent. Participants aged 18 years or older who met any of the following criteria were included:

(1) Patients diagnosed with urothelial carcinoma of the bladder (UCB) and upper tract urothelial carcinoma (UTUC) via cystoscopy, ureteroscopy, and preoperative biopsy histopathology.

(2) Patients with common benign urologic conditions (such as urinary stones, inflammation, ureteral stenosis, cysts, and benign prostatic hyperplasia), as well as those with other urologic tumors (such as kidney cancer and prostate cancer).

(3) Healthy individuals undergoing routine medical examinations.

The exclusion criteria were:

(1) Patients with urothelial carcinoma (UC) concurrently diagnosed with other malignant tumors.

(2) Patients who had received chemotherapy or radical treatment for UC in the past six months.

(3) Urine samples with low DNA concentration or those that failed quality control tests.

(4) Patients with missing clinical data.

In the training cohort, we recruited 39 UC patients, 5 patients with other tumors, and 54 patients with benign urological diseases from Zhujiang Hospital of Southern Medical University between March 2022 and November 2022. In the prospective and blinded cohort, we recruited 189 UC patients, 30 patients with other tumors, and 136 patients with benign urological diseases from two additional hospitals in China. Patients in the multicenter validation cohort were recruited from Peking University First Hospital (validation cohort 1, n = 245) and Meizhou People's Hospital (validation cohort 2, n = 110). Clinical and pathological characteristics of patients were shown in **Table S1**. We classified patients with other tumors and benign urological diseases as the control group. During this study, blinding was implemented for the operators performing the diagnostic panel and for the evaluators comparing the results to the reference method (pathology). This measure ensured that they were unaware of the subjects' disease diagnoses or any other relevant test results during the experimental process.

**Sample processing and DNA isolation**

For all participants, a midstream morning urine sample of at least 30 mL was collected and mixed with a specific urine preservation solution. The samples were centrifuged at room temperature at 3000 × *g* for 10 minutes. After centrifugation, the supernatant was discarded, and the pellet was resuspended in PBS. The resuspended pellet was carefully transferred to new 2 mL tubes, centrifuged again, and the supernatant was discarded. The final pellet was stored at -80°C. Genomic DNA was extracted using the Tissue Genomic DNA Extraction Kit (cat DP304, Tiangen Biotechnology, Beijing, China) according to the manufacturer's instructions.

**Methylation analysis**

100 ng of genomic DNA was subjected to bisulfite conversion and purification using the EZ DNA Methylation-Lightning™ Kit (Zymo Research Corporation, Irvine, CA, USA) according to the manufacturer's protocol. During bisulfite conversion, unmethylated cytosines were deaminated to uracil, while methylated cytosines remained unchanged. The bisulfite-converted DNA was then subjected to duplex PCR amplification. Methylated *NRN1* gene regions were amplified using specific primers, with methylation detected via fluorescence released from FAM-labeled probes during *Taq* enzyme activity. Concurrently, specific primers were used to amplify the internal reference gene *ACTB* from the bisulfite-converted template, with VIC-labeled probes detecting *ACTB* to assess DNA quantity and quality. The sequences of the primers used are as follows:

*NRN1* forward: GTTTGTAAATTGTTGTAGGAAGCGC,

reverse: ATATTCGCTATAAAATAAACCCG,

probe: ATCGAAATTAACCTCGATCCCTTCC;

*ACTB* forward: AGTGAGAAAGGGTGTAGTTTTGGGAG,

reverse: CCACAAAAAAATAACCCAAATAAATAACCCACT,

probe: CCTCTTCTAATAACCACCTCCCTCCTTCCTAAC.

**Statistical analysis**

The receiver-operating characteristic (ROC) curve was constructed and the area under the curve (AUC) was plotted to evaluate the model performance. Sensitivity and specificity were calculated, demonstrating the diagnostic accuracy of the *NRN1* methylation test. The *p* values are from two-sided statistical tests, and differences with *p* ≤ 0.05 were considered significant. All statistical analyses and data visualizations were carried out using SPSS software (version 26.0, SPSS Inc., Chicago, IL, USA), R software (version 4.2.1), and Prism 10 (GraphPad Software).


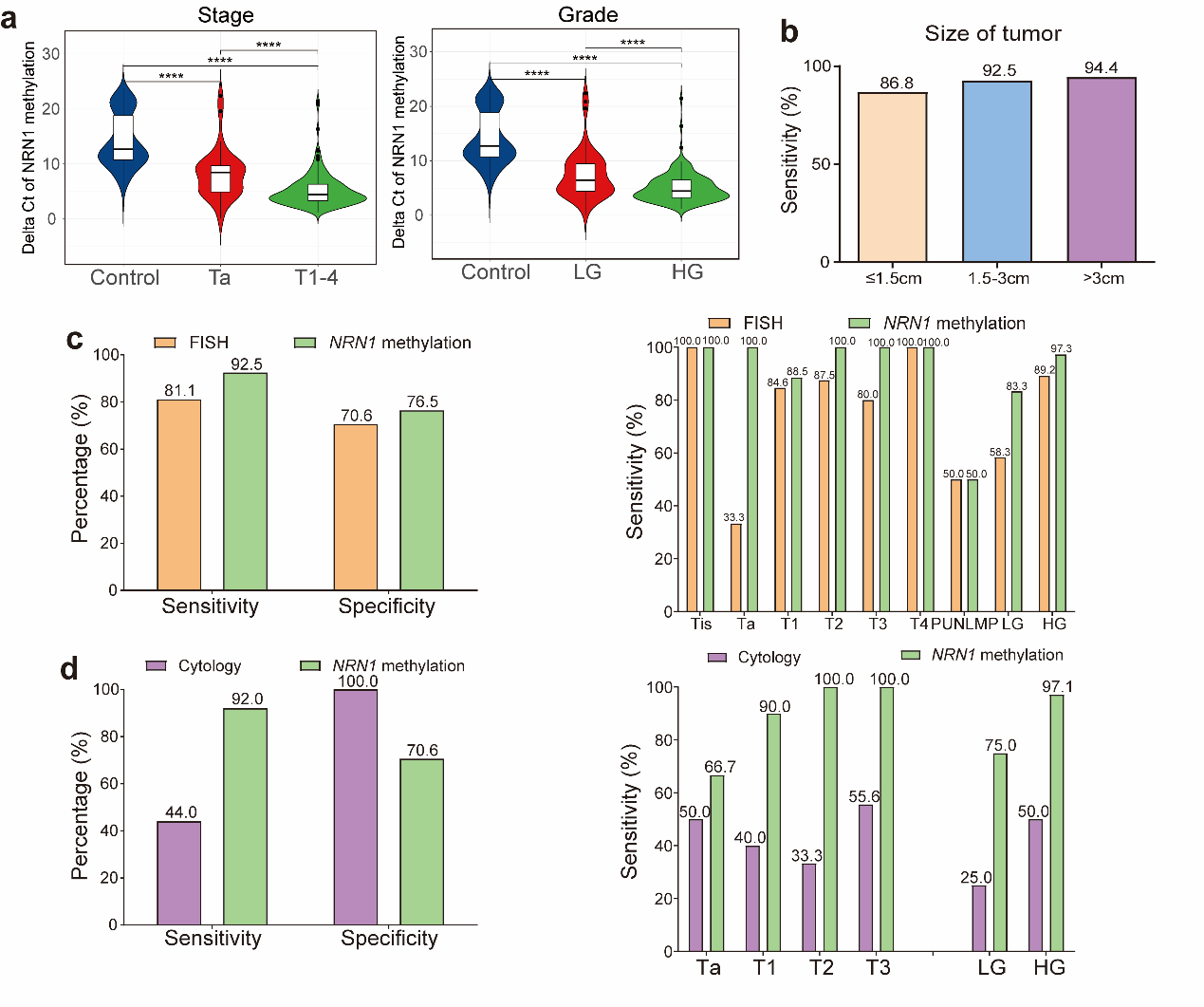


**Figure S1** Subgroup analysis and comparison of the *NRN1*^me^ test for UC prediction. **a** *NRN1* methylation levels across different stages and grades of UC patients. **b** Sensitivity of the *NRN1*^me^ test across different tumor size groups. **c** Comparison of sensitivity and specificity for UC prediction between FISH and the *NRN1*^me^ test, as well as sensitivity analyses across different stage and grade subgroups. **d** Comparison of sensitivity and specificity for UC prediction between cytology and the *NRN1*^me^ test, as well as sensitivity analyses across different stage and grade subgroups. UC, urothelial carcinoma; UCB, urothelial carcinoma of bladder; UTUC, upper tract urothelial carcinoma; LG, low grade; HG, high grade; PUNLMP, papillary urothelial neoplasm of low malignant potential. *****p* < 0.0001.
